# Association between convalescent plasma and the risk of mortality among patients with COVID-19: a meta-analysis

**DOI:** 10.12688/f1000research.36396.1

**Published:** 2021-02-03

**Authors:** Shinta Oktya Wardhani, Jonny Karunia Fajar, Laksmi Wulandari, Gatot Soegiarto, Yeni Purnamasari, Anisa Asmiragani, Helnida Anggun Maliga, Muhammad Ilmawan, Gloriana Seran, Dheka Sapti Iskandar, Conchita Emiliana Ndapa, Viviana Hamat, Rafika Ajeng Wahyuni, Linda Oktaviana Suci Cyntia, Feronika Maryanti Maarang, Yosef Andrian Beo, Olivera Agnes Adar, Iraky Mardya Rakhmadhan, Emilia Tiara Shantikaratri, Ayu Sekarani Damana Putri, Rizqa Wahdini, Endang Pati Broto, Agnes Wanda Suwanto, Fredo Tamara, Aditya Indra Mahendra, Eden Suryoiman Winoto, Pratista Adi Krisna, Harapan Harapan

**Affiliations:** 1 Division of Hematology and Oncology, Department of Internal Medicine, Faculty of Medicine, Universitas Brawijaya, Malang, 65145, Indonesia; 2 Brawijaya Internal Medicine Research Center, Department of Internal Medicine, Faculty of Medicine, Universitas Brawijaya, Malang, 65145, Indonesia; 3 Department of Pulmonology and Respiratory Medicine, Faculty of Medicine, Universitas Airlangga, Surabaya, 60286, Indonesia; 4 Division of Allergy & Immunology, Department of Internal Medicine, Faculty of Medicine, Universitas Airlangga, Surabaya, 60286, Indonesia; 5 Faculty of Medicine, Universitas Brawijaya, Malang, 65145, Indonesia; 6 Faculty of Medicine, Universitas Indonesia, Jakarta, 10430, Indonesia; 7 Department of Biomedical Sciences, Faculty of Medicine, Universitas Brawijaya, Malang, 65145, Indonesia; 8 Department of Midwifery, Faculty of Medicine, University Brawijaya, Malang, 65145, Indonesia; 9 Department of Nursing, Faculty of Medicine, Universitas Brawijaya, Malang, 65145, Indonesia; 10 Department of Neurosurgery, Faculty of Medicine, Universitas Airlangga, Surabaya, 60286, Indonesia; 11 Department of Radiology, Faculty of Medicine, Universitas Brawijaya, Malang, 65145, Indonesia; 12 Medical Research Unit, School of Medicine, Universitas Syiah Kuala, Banda Aceh, 23111, Indonesia

**Keywords:** convalescent plasma, passive immunization, COVID-19, mortality, outcomes

## Abstract

**Background:** Convalescent plasma (CCP) has been used for treating some infectious diseases; however, the efficacy of CCP in coronavirus disease 2019 (COVID-19) remains controversial. The aim of this research was to assess the efficacy of CCP as an adjunctive treatment in COVID-19 patients.

**Methods:** Four bibliographic databases and a preprint database were searched for potentially relevant articles. Mortality rates between patients treated with standard treatment and standard treatment with CCP were compared using a Z test.

**Results:** A total of 1,937 patients treated with CCP and 3,405 patients without CCP retrieved from 12 studies were included. The risk of mortality was 1.92-fold higher in patients without CCP compared to patients treated with CCP (OR: 1.92; 95%CI: 1.33, 2.77; p=0.0005). In severe COVID-19 sub-group analysis, we found that patients without the CCP had a 1.32 times higher risk of mortality than those treated with the CCP (OR: 1.32; 95%CI: 1.09, 1.60; p=0.0040).

**Conclusions:** CCP, as adjunctive therapy, reduces the mortality rate among COVID-19 patients.

## Introduction

The management of coronavirus disease 2019 (COVID-19) remains challenging. While the guideline for the management of COVID-19 has been established, ^1- 3^ the mortality rate of COVID-19 remains increased over the periods. ^4, 5^ The guideline suggests that several treatments, including antiviral, hydroxychloroquine, steroid, anticoagulation, and other supportive treatments, should be used to treat patients with COVID-19. ^1- 3^ However, recent evidence from large scale studies failed to clarify the efficacy of those suggested treatments. ^6- 8^ Moreover, the findings from the World Health Organization (WHO) solidarity trials also failed to clarify the benefits of hydroxychloroquine, remdesivir, interferon regimens, and lopinavir in the management of COVID-19. ^8^ Therefore, new approaches to COVID-19 management are required.

Convalescent plasma (CCP), an immunological therapy, is suggested to have promising efficacy for managing several infectious diseases. ^9^ CCP, a strategy of passive immunization, was first introduced by von Behring and Kitasato in 1890. Initially, it was used to manage diphtheria and other infectious diseases such as scarlet fever and pertussis. ^10^ Moreover, due to its good efficacy, this therapy was also used for the management of Ebola, severe acute respiratory syndrome (SARS), and Middle East respiratory syndrome (MERS). ^11^ In patients with MERS, SARS, and Ebola, the clinical improvement and reduced mortality rate were observed in patients receiving CCP than patients without CPP. ^12^ However, the efficacy of CCP against COVID-19 is conflicting. Furthermore, previous meta-analyses resulted in inconclusive findings due to the lack of structured methodology. Therefore, a holistic meta-analysis is needed to provide insight into the clinical efficacy of CCP for the management of COVID-19.

## Methods

### Study design

A systematic review and meta-analysis covering the period July 2020 - December 2020 was conducted to assess the efficacy of CCP as an adjunctive treatment in COVID-19 patients. Studies from prominent bibliographic databases were searched, and the protocols followed the checklist from Preferred Reporting Items for Systematic Review and Meta-analysis (PRISMA). ^13^

### Eligibility criteria

Relevant articles were assessed for inclusion and exclusion criteria before the final analysis. Our analysis included articles with the following criteria: (1) observational or randomized controlled trial studies; (2) providing sufficient data of COVID-19 diagnosis methods; and (3) well-identified methodologies represented with Newcastle-Ottawa Scale (NOS). Case reports, case series, letters to the editor, reviews, commentaries, low method quality, and those with pre-post test comparison were excluded.

### Search strategy and data extraction

Relevant studies in four bibliographic databases (Embase, PubMed, Web of Science, and Cochrane) and a preprint database MedRix were searched as of 2 December 2020. The searches limited to English only using Medical Subjects Heading: (“COVID-19” OR “SARS-CoV-2”) AND (“convalescent plasma” OR “serotherapy” OR “hyperimmune globulin therapy” OR “convalescent plasma treatment”). A reference list of the relevant articles was also retrieved for additional references. Information of: (1) name of the first author; (2) year of publication; (3) country of origin; (4) sample size of cases and controls, (5) CCP administration, and (6) mortality rate were collected from each article.

### Assessment of the methodology quality

The quality of potential papers was assessed using the Newcastle-Ottawa Scale (NOS). ^14^ This scoring system evaluates the sample selection, group comparison, and the outcome. The quality of the articles could be classified as low, moderate, and high quality. Articles with low quality were excluded from our analysis. The assessment was carried out by three independent investigators (MI, AAA & YP), and when there was a discrepancy among the investigators, a discussion was performed with a senior researcher (JKF).

### Outcome measure

The primary outcome measure was all causes of mortality among COVID-19 patients treated with and without CCP. The predictor variable was COVID-19 patients treated with CCP. A sub-group analysis was conducted based on the severity of COVID-19 patients treated with CCP (e.i. mild and severe).

### Statistical analysis

The association between CCP and the reduction of the risk of mortality among COVID-19 patients was assessed using a Z test. Before assessing the association, the potency of bias and heterogeneity was assessed. To assess the risk of bias, an Egger test was employed to calculate tau-squared, and a p-value of less than 0.05 indicates that the potency of bias was found. A Q test was used to assess the heterogeneity among the included papers. The p-value of less than 0.10 was considered that heterogeneity across the studies was found, and the correlation was therefore determined using a random-effect model; otherwise, a fixed-effect model was employed. All analyses were carried out using Review Manager (Revman Cochrane, London, UK) version 5.3, and the cumulative calculation was presented using a forest plot.

## Results

### Studies selection

**Figure 1.**
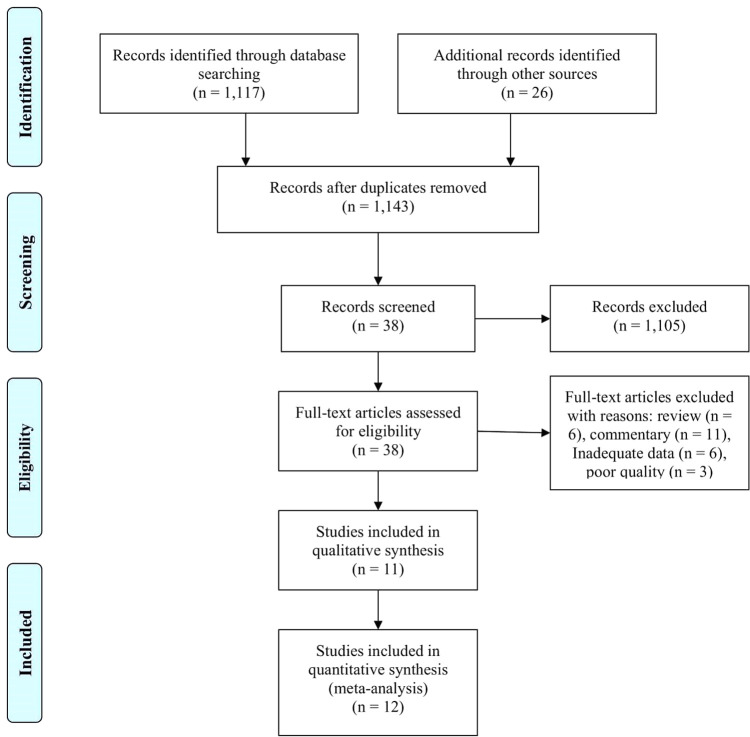
A flowchart of study selection in our meta-analysis.

A total of 1,143 papers were identified, and 1,105 papers were excluded because they had irrelevant topics. A total of 38 papers were included for review in full-text, and 26 additional papers were excluded because of review, pre-post test model, commentary, and low-quality papers. In the final process, 12 papers were included in our analysis. ^15- 26^ The article selection flowchart is depicted in Figure 1, and the study characteristics are presented in Table 1.

### CCP efficacy against COVID-19

**Figure 2.**
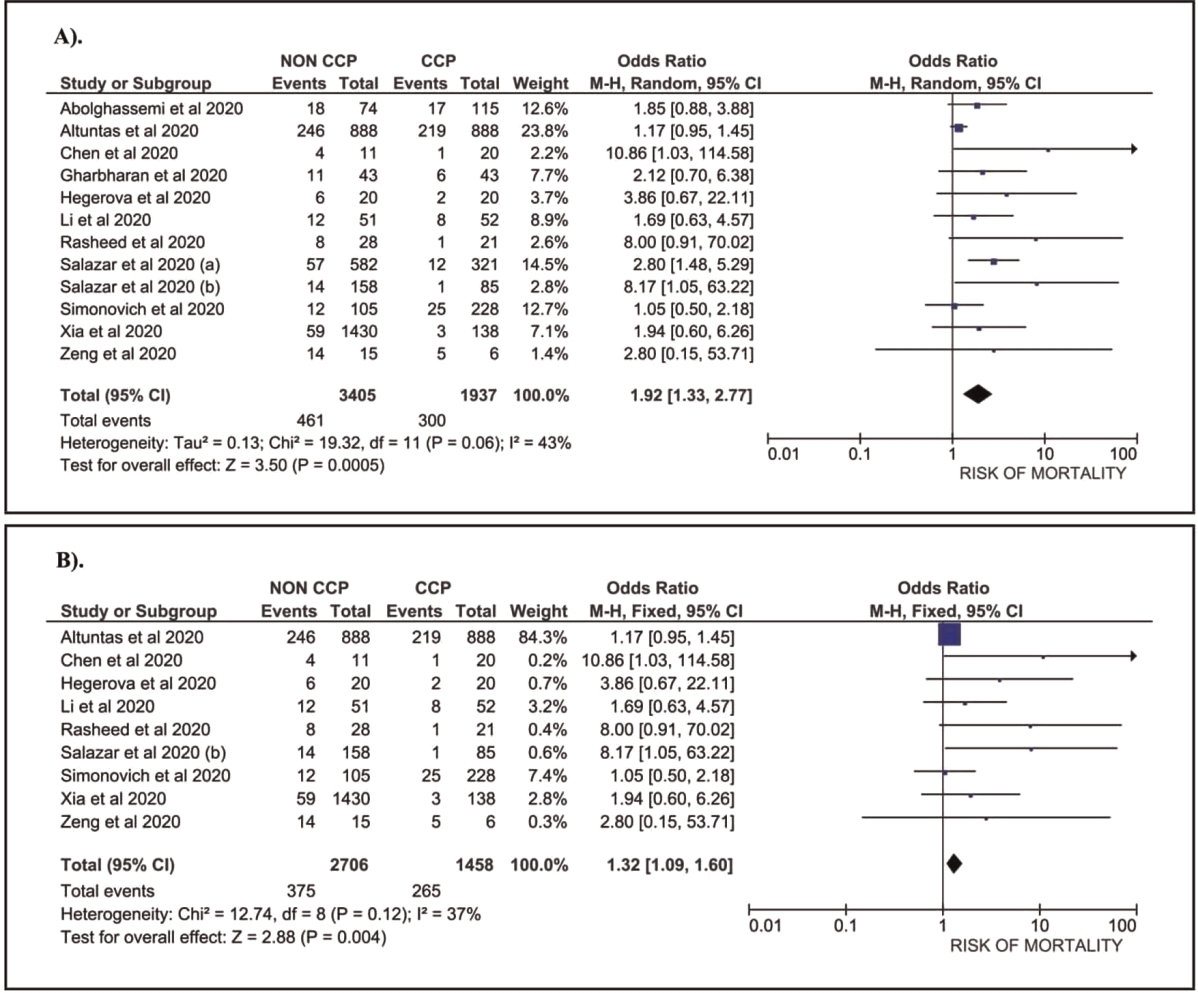
Forest plot of the association between convalescent plasma and the risk of mortality. A). All cases (OR: 1.92; 95%CI: 1.33, 2.77; p = 0.0005; p Egger: 0.3620; p Heterogeneity: 0.0600; I-squared: 43.00%). B). Severe COVID-19 (OR: 1.32; 95%CI: 1.09, 1.60; p = 0.0040; p Egger: 0.3790; p Heterogeneity: 0.1200; I-squared: 37.00%).

A total of 1,937 patients treated with CCP and 3,405 patients without CCP, collected from 12 papers, were included in our analysis. Data suggest that COVID-19 patients without the CCP had a 1.92-fold higher risk of mortality than patients treated with the CCP (OR: 1.92; 95%CI: 1.33, 2.77; p=0.0005) ( Figure 2A). A sub-group analysis among severe COVID-19 patients who were treated with CCP was conducted. This sub-group consisted of nine papers with 1,458 patients treated with CCP and 2,706 patients without CCP. The data revealed a 1.32-fold higher risk of mortality in COVID-19 patients without CCP compared to patients treated with CCP (OR: 1.32; 95%CI: 1.09, 1.60; p=0.0040) ( Figure 2B).

**Table 1.** Baseline characteristics of articles included in our meta-analysis.

Name	Country	City	Hospital	Sample size		CCP volume	Recipient	NOS
				CCP	Control			
Abolghassemi et al 2020	Iran	Mixed	Multicenter	115	74	500 mL	Mild and severe cases	9
Altuntas et al 2020	Turkey	Mixed	Multicenter	888	888	200 - 600 mL	Severe cases	8
Chen et al 2020	China	Hangzhou	Zhejiang University	19	10	200-500 mL	Severe cases	7
Gharbharan et al 2020	Netherlands	Mixed	Multicenter	43	43	300 mL	Mild and severe cases	8
Hegerova et al 2020	USA	Washington	Multicenter	20	20	200 mL	Severe cases	9
Li et al 2020	China	Wuhan	Multicenter	52	51	100 mL	Severe cases	8
Rasheed et al 2020	Iraq	Bagdad	Multicenter	21	28	400 mL	Severe cases	8
Salazar et al 2020 (a)	US	Mixed	Multicenter	321	582	NA	Mild and severe cases	8
Salazar et al 2020 (b)	US	Mixed	Multicenter	85	158	NA	Severe cases	9
Xia et al 2020	China	Wuhan	Wuhan Huoshenshan Hospital	138	1430	200-1200 mL	Severe cases	8
Zeng et al 2020	China	Hangzhou	Multicenter	6	15	300 mL	Severe cases	9

Note: CCP, convalescent plasma; NOS, Newcastle-ottawa scale.

### Heterogeneity and potency of bias across the studies

The analysis revealed evidence of heterogeneity in total case of COVID-19. Therefore, a random-effect model was applied to assess the association. In the severe COVID-19 sub-group, we found no heterogeneity, and we used a fixed-effect model to evaluate the correlation. Our analysis using an Egger test found no publication bias in both the total and the severe COVID-19 sub-group.

## Discussion

Our data suggest that CCP treatment associated with a reduction of mortality both in all cases and severe COVID-19 patients. Our current findings are consistent with the results of previous meta-analyses. ^27- 32^ The theory underlying the mechanism of CCP in COVID-19 patients remains open to controversy. Briefly, plasma transfer is the potential aspect that bridges the CCP and the reduced risk of mortality in COVID-19 patients. Plasma consists of various immunity components, including antibodies, anti-inflammatory cytokines, clotting and or anti-clotting factors, albumin, and protein C and S. ^33, 34^ It is believed that CCP in COVID-19 may modulate the immune response through antiviral effects and has immunomodulatory effects. ^35^ Antiviral effects of CCP may occur through neutralizing antibodies, and it was reported that IgG of severe acute respiratory syndrome coronavirus 2 (SARS-CoV-2) and IgM SARS-CoV-2 were the primary isotype antibodies identified from COVID-19 patients treated with CCP. ^36^ This humoral immune response may inhibit protein S of SARS-CoV-2. ^37^ Thereafter, they may exert the protective effects against COVID-19. The immunomodulatory effects of CCP may occur through the neutralization of cytokines and complements. ^35, 38^ These effects may inhibit the overactive immune system, including cytokine storm, complement activation, and hypercoagulable state regulation. ^39^ These mechanisms may be responsible for causing clinical improvement of COVID-19 patients. Of them, it was considered that immunoglobulin transfer is the essential factor in modulating the protective effect of CCP. ^40^ In SARS and influenza, it was reported that immunoglobulin transfer plays a vital role in governing clinical improvement. ^9, 11^ Moreover, in MERS, the CCP administration with the titers of antibodies 1:80 provided a significant immune response, and the titers of antibodies 1:40 did not provide a similar response. ^41^ Additionally, in Ebola, MERS, and SARS, the antibodies from the CCP may bind to the CD4 binding site on the viral envelope, and therefore may reduce the viral load and the risk of infection of the new cells. ^42^ It was also supported by previous studies that antibody titers from CCP donors also governed the clinical improvement of COVID-19 patients treated with CCP, ^43, 44^ suggesting that antibody transfer might influence the outcomes of clinical improvement.

Six meta-analyses assessing the role of CCP in COVID-19 have been reported ( Table 2). ^27- 32^ However, they had some significant limitations: (a) they involved a smaller sample size. In our current study, we had a relatively larger sample size; (b) some studies did not perform meta-analysis calculations to synthesize the data ^27, 29^; (c) previous studies included several case reports and case series ^28, 29^ in which should be excluded in the meta-analysis ^13^; (d) previous meta-analyses assessed the role of CCP in similar infectious diseases (SARS and influenza), and the results were implemented to the case of COVID-19 ^30, 31^; and (e) previous meta-analyses performed a mixed calculation where the data of the case vs. control model were combined with the data of pre-post intervention models, which might provide a high risk of bias due to the final effect that might be caused by other interventions. ^29, 32^ In the present meta-analysis, we only calculated the model of the case (standard treatment and CCP) vs. control (standard treatment only) and therefore might provide a better correlation.

**Table 2.** Previous meta-analyses and some potential limitations.

Author & year	Number of studies	Sample size	Potential limitations
Bakhtawar et al 2020	10	156	- No calculation of data synthesis - Seven case report or case series articles were included - One study comparing the outcome between pre and post convalescent plasma.
Devasenapathy et al 2020	6	431	- The case is non COVID-19
Rabelo-da-Ponte et al 2020	5	75	- Three case report or case series articles were included - The comparison was pre and post convalescent plasma.
Rajendran et al 2020	5	NA	- No calculation of data synthesis
Sarkar et al 2020	7	5444	- One study comparing the outcome between pre and post convalescent plasma, other studies assessing between convalescent plasma and control (Mixed calculation). - Inappropriate calculation.
Sun et al 2020	15	1879	- The case is non COVID-19

Note: NA, Not available; CCP, convalescent plasma.

In the present study, we emphasized that CCP provided good efficacy to reduce the risk of mortality among COVID-19 patients. Our findings might contribute to better management of COVID-19 patients, particularly to prevent the risk of mortality. It is expected that a medical council should elaborate on the standard procedures of CCP, including the dosage, donor criteria, side effects management, and post-intervention management. Since early administration of CCP provided better clinical outcomes than those with later intervention, ^45^ the appropriate time of CCP administration should be determined, and further studies are warranted.

Several important limitations of this study should be discussed. Some confounding factors that might govern the final outcomes were not controlled, including the immunological status, the dosage of CCP, time of intervention, donor criteria, the titers of antibodies, comorbidities, and transmission area. The majority of the included papers were retrospective studies, and therefore a further meta-analysis of randomized-controlled trials with a bigger sample size might provide a better conclusion.

## Conclusion

Administration of the CCP is associated with a lower risk of mortality among COVID-19 patients compared to those without CCP, and this highlights its potency to be used for the treatment of COVID-19. However, studies are warranted to formulate the dosage, time of intervention, donor criteria, and the titers of antibodies to optimize the effects.

## Data availability

### Underlying data

All data underlying the results are available as part of the article and no additional source data are required.

### Reporting guidelines

Figshare: PRISMA checklist for ‘Association between convalescent plasma and the risk of mortality among patients with COVID-19: A meta-analysis’, https://doi.org/10.6084/m9.figshare.13490541.v1. ^46^

Data are available under the terms of the Creative Commons Attribution 4.0 International license (CC-BY 4.0).
