# Supplementary material for: Association between convalescent plasma and the risk of mortality among patients with COVID-19: a meta-analysis
Source: F1000Res. 2021 Jun 2;10:64. Originally published 2021 Feb 3. [Version 3] doi: 10.12688/f1000research.36396.3 (PMC8182694; doi:10.12688/f1000research.36396.3)
Supplement: Peer review history

# Peer review: Reviewer response for version 3

**DOI:** 10.5256/f1000research.55951.r86549

**Published:** 2021-06-04

**Authors:** Guilherme Welter Wendt

**Affiliations:** 1 Health Sciences Center, Western Paraná State University (UNIOESTE), Francisco Beltrão, Brazil

After reading the revised version of the paper, I can see that authors addressed the previous suggestions and their manuscript seems suitable for appproval.

Is the work clearly and accurately presented and does it cite the current literature?

Yes

If applicable, is the statistical analysis and its interpretation appropriate?

Yes

Are all the source data underlying the results available to ensure full reproducibility?

No source data required

Is the study design appropriate and is the work technically sound?

Yes

Are the conclusions drawn adequately supported by the results?

Yes

Are sufficient details of methods and analysis provided to allow replication by others?

Partly

Reviewer Expertise:

Quantitative research methods; systematic reviews;  meta-analyses

I confirm that I have read this submission and believe that I have an appropriate level of expertise to confirm that it is of an acceptable scientific standard.

# Peer review: Reviewer response for version 2

**DOI:** 10.5256/f1000research.54795.r81757

**Published:** 2021-03-29

**Authors:** Guilherme Welter Wendt

**Affiliations:** 1 Health Sciences Center, Western Paraná State University (UNIOESTE), Francisco Beltrão, Brazil

The study sought to assess the efficacy of CCP as an adjunctive treatment in COVID-19 patients. The authors are encouraged to:

1. Correctly report the names of the instruments used (Newcastle-Ottawa, and not Newcaste-ottawa; Jadad scale, and not jadad) in the whole text, including the abstract.
2. There are two dots in the end of the second sentence of the results section in the abstract.
3. In the introduction, the following sentence could be expanded: “the mortality rate of COVID-19 remains increased over the periods”. Please, focus on the literature you cited and be specific as possible. For instance, some reports show the opposite, that is, that treatment has improved so the mortality rate has decreased. The authors’ very own findings point to lower OR for death among those treated with CCP.
4. Albeit it is true that the solidarity trial showed little efficacy of the drugs mentioned in the introduction, a robust finding was found in severe patients that were treated with dexamethasone.
5. Would you please give more information on clinical improvement of the diseases treated with CCP cited in the second paragraph (introduction, ref. n. 12)?
6. The following sentence needs a few supporting references: “However, the efficacy of CCP against COVID-19 is conflicting”.
7. I would suggest the authors to remove the word “holistic” when presenting their goal.
8. When presenting the eligibility criteria, I was wondering why pre-post comparisons were excluded. Would be worth it to justify this choice.
9.  I would change this sentence “If the disagreement was found, we performed a discussion to resolve the disagreement” into “Disagreements were resolved in group discussions”. Also, did you ask for an external judge to assist in the discussion of disagreements? Or a consensus approach was used? This is unclear.
10. There is a typo in the section describing the outcome variable (e.i. mild and severe). Please, correct to “i.e.”; Also, the outcome variables could be more clear. Do you mean “the number or COVID-19 patients treated with CCP”?
11. In the section “Heterogeneity and potency of bias across the studies”, please be clear/complete in the following sentence “a random-effect model was applied to assess the Association…”. Association between what?
12. This sentence needs more information: “In the present meta-analysis, we only calculated the model of the case (standard treatment and CCP) vs. control (standard treatment only) and therefore might provide a better correlation”. Would you please justify why your approach provides better ‘correlation’?
13. Please, double check the comprehensiveness of Table 2. The table is supposed to present previous meta-analyses and some of the studies included there were judged to not calculate data synthesis. However, I see that some studies are only systematic reviews, such as Rajendran and collaborators.
14. Supplementary file 2 has some typos ( https://figshare.com/articles/dataset/SUPPLEMENTARY_FILE_2_THE_ASSOCIATION_BETWEEN_CONVALESCENT_PLASMA_AND_THE_RISK_OF_MORTALITY/14046254/1), including “Funnel plot of tha association”. Would you please correct it?
15. The supplementary Prisma checklist contains information not covered in the study. For instance, the authors said that the systematic review registration number has been given in the abstract, albeit I was not able to find it. This checklist also asks the authors to be explicit about the language of papers under “eligibility”. This should be made clear in the text and in the supplementary checklist. Item 19 of the same checklist asks that authors “present data on risk of bias of each study”. I could not locate this in the text. Please, revise this item.

Is the work clearly and accurately presented and does it cite the current literature?

Yes

If applicable, is the statistical analysis and its interpretation appropriate?

Yes

Are all the source data underlying the results available to ensure full reproducibility?

No source data required

Is the study design appropriate and is the work technically sound?

Yes

Are the conclusions drawn adequately supported by the results?

Yes

Are sufficient details of methods and analysis provided to allow replication by others?

Partly

Reviewer Expertise:

Quantitative research methods; systematic reviews;  meta-analyses

I confirm that I have read this submission and believe that I have an appropriate level of expertise to confirm that it is of an acceptable scientific standard, however I have significant reservations, as outlined above.

# Response

**Published:** 2021-04-06

**Authors:** Jonny Fajar

**Affiliations:** Brawijaya Internal Medicine Research Center, Department of Internal Medicine, Faculty of Medicine, Universitas Brawijaya, Malang, Indonesia, Indonesia

**Response to reviewer comments:**

 The study sought to assess the efficacy of CCP as an adjunctive treatment in COVID-19 patients. The authors are encouraged to:

 Correctly report the names of the instruments used (Newcastle-Ottawa, and not Newcaste-ottawa; Jadad scale, and not jadad) in the whole text, including the abstract.

**Response:** The phrase has been revised.

 There are two dots in the end of the second sentence of the results section in the abstract.

**Response: **We have revised the sentence.

 In the introduction, the following sentence could be expanded: “the mortality rate of COVID-19 remains increased over the periods”. Please, focus on the literature you cited and be specific as possible. For instance, some reports show the opposite, that is, that treatment has improved so the mortality rate has decreased. The authors’ very own findings point to lower OR for death among those treated with CCP.

**Response:** The sentence has been revised.

 Albeit it is true that the solidarity trial showed little efficacy of the drugs mentioned in the introduction, a robust finding was found in severe patients that were treated with dexamethasone.

**Response:** We only reported the findings of the trial. The trial did not include dexametasone.

 Would you please give more information on clinical improvement of the diseases treated with CCP cited in the second paragraph (introduction, ref. n. 12)?

**Response: **The clinical improvement indicated the improvement of disease manifestation.

 The following sentence needs a few supporting references: “However, the efficacy of CCP against COVID-19 is conflicting”.

**Response:** In this sentence, we tried to explained that, among all included studies in our analysis, some studies found the efficacy of CCP for treating COVID-19, while other studies failed to clarify the efficacy. The references were provided in the results section.

 I would suggest the authors to remove the word “holistic” when presenting their goal.

**Response: **the word “holistic” has been removed.

 When presenting the eligibility criteria, I was wondering why pre-post comparisons were excluded. Would be worth it to justify this choice.

**Response: **The reason why pre-post comparisons were excluded from our study is we considered that the outcomes of therapy might have high risk of bias due to the final outcomes were affected by CCP or other medications.

 I would change this sentence “If the disagreement was found, we performed a discussion to resolve the disagreement” into “Disagreements were resolved in group discussions”. Also, did you ask for an external judge to assist in the discussion of disagreements? Or a consensus approach was used? This is unclear.

**Response:** The sentence has been revised.

 There is a typo in the section describing the outcome variable (e.i. mild and severe). Please, correct to “i.e.”; Also, the outcome variables could be more clear. Do you mean “the number or COVID-19 patients treated with CCP”?

**Response: **The sentence has been revised.

 In the section “Heterogeneity and potency of bias across the studies”, please be clear/complete in the following sentence “a random-effect model was applied to assess the Association…”. Association between what?

**Response: **The sentence has been revised.

 This sentence needs more information: “In the present meta-analysis, we only calculated the model of the case (standard treatment and CCP) vs. control (standard treatment only) and therefore might provide a better correlation”. Would you please justify why your approach provides better ‘correlation’?

**Response:** We considered that the design of standard treatment and CCP vs. standard treatment only, and the patients were followed up after the periods might have better efficacy than the design of pre-post comparisons. In the design of  pre-post comparisons, the outcomes of therapy might have high risk of bias due to the final outcomes were affected by CCP or other medications.

 Please, double check the comprehensiveness of Table 2. The table is supposed to present previous meta-analyses and some of the studies included there were judged to not calculate data synthesis. However, I see that some studies are only systematic reviews, such as Rajendran and collaborators.

**Response:** The sentence has been revised.

 Supplementary file 2 has some typos (https://figshare.com/articles/dataset/SUPPLEMENTARY_FILE_2_THE_ASSOCIATION_BETWEEN_CONVALESCENT_PLASMA_AND_THE_RISK_OF_MORTALITY/14046254/1), including “Funnel plot of tha association”. Would you please correct it?

**Response: **The supplementary file 2 has been revised.

 The supplementary Prisma checklist contains information not covered in the study. For instance, the authors said that the systematic review registration number has been given in the abstract, albeit I was not able to find it. This checklist also asks the authors to be explicit about the language of papers under “eligibility”. This should be made clear in the text and in the supplementary checklist. Item 19 of the same checklist asks that authors “present data on risk of bias of each study”. I could not locate this in the text. Please, revise this item.

**Response:** The study registration is on process. The risk of bias was assessed using Egger test. The quality of each study was assessed using Newcastle-ottawa scale and Modified Jadad scale.

# Peer review: Reviewer response for version 2

**DOI:** 10.5256/f1000research.54795.r81042

**Published:** 2021-03-09

**Authors:** Morteza Arab-Zozani

**Affiliations:** 1 Social Determinants of Health Research Center, Birjand University of Medical Sciences, Birjand, Iran

Thank you for clearly addressing my previous comments. In my opinion, the manuscript is acceptable in this way.

Is the work clearly and accurately presented and does it cite the current literature?

Partly

If applicable, is the statistical analysis and its interpretation appropriate?

Partly

Are all the source data underlying the results available to ensure full reproducibility?

Yes

Is the study design appropriate and is the work technically sound?

Partly

Are the conclusions drawn adequately supported by the results?

No

Are sufficient details of methods and analysis provided to allow replication by others?

Partly

Reviewer Expertise:

Health-related issues and systematic review and meta-analysis methodology.

I confirm that I have read this submission and believe that I have an appropriate level of expertise to confirm that it is of an acceptable scientific standard.

# Peer review: Reviewer response for version 1

**DOI:** 10.5256/f1000research.39458.r78924

**Published:** 2021-02-15

**Authors:** Morteza Arab-Zozani

**Affiliations:** 1 Social Determinants of Health Research Center, Birjand University of Medical Sciences, Birjand, Iran

This meta-analysis aimed to investigate the association between convalescent plasma and the risk of mortality among patients with COVID-19. This is a great area of research but, in my opinion, the manuscript needs some major revisions as follows:

- Please indicate the name of the searched databases in the abstract section.
- Please indicate the quality appraisal checklist in the abstract section.
- Please indicate the method for investigating the heterogeneity and publication bias in the abstract section. 
- Please indicate the type of the included studies in the abstract, results, and table 1.
- What is your reason for selecting this period for your search?
- Search strategy is not complete. 
- Please restructure the method section following the PRISMA item as you claim.
- There are some problems regarding figure 1. Was there no duplicate record? It does not make much sense. 
- It needs to mention the type of the included studies and then we can speak about the quality appraisal checklist. it seems that NOS is not sufficient. NOS is for nonrandomized studies.
- Please indicate inter-rater reliability between three raters. 
- Result section, please add a subheading for "study characteristics" based on PRISMA and first write a brief and then refer to table 1. Also, add the type of the control in column control. 
- Figure 1 has some problems. Your study is a meta-analysis. How were 11 studies included in qualitative synthesis? Which qualitative synthesis?
- There is a 6 % difference between I2 for A and B, what is your rationale for selecting the fixed or random-effect model? Please provide a reference for your claim. Please add the details in the method section.
- Please add the funnel plot as a supplement.
- Please remove table 2 from the discussion and also discuss the added value of your study regarding the existing meta-analysis. What is the novelty of your work?
- The conclusion is very optimistic. How did you come to that conclusion based solely on mortality?

Is the work clearly and accurately presented and does it cite the current literature?

Partly

If applicable, is the statistical analysis and its interpretation appropriate?

Partly

Are all the source data underlying the results available to ensure full reproducibility?

Yes

Is the study design appropriate and is the work technically sound?

Partly

Are the conclusions drawn adequately supported by the results?

No

Are sufficient details of methods and analysis provided to allow replication by others?

Partly

Reviewer Expertise:

Health-related issues and systematic review and meta-analysis methodology.

I confirm that I have read this submission and believe that I have an appropriate level of expertise to state that I do not consider it to be of an acceptable scientific standard, for reasons outlined above.
